# Quack Advertisements in Religious Newspapers

**Published:** 1888-03

**Authors:** 


### QUACK ADVERTISEMENTS IN RELIGIOUS NEWSPAPERS.

The *Medical and Surgical Reporter* very sensibly remarks: "From time to time medical men and medical journals have protested against the prostitution of the columns of religious newspapers to the use of advertisers of quack nostrums. This protest does not apply to temperately worded representations of what seems to have been accomplished by, or what may reasonably be expected of, a remedy or device for the cure of disease or injury. But it does apply to advertisements couched in language which bears the stamp of falsehood on its face, or which is of such a character as to arouse suspicion in the mind of an intelligent man, uninfluenced by a money consideration.

"The editors of most religious journals are, as a rule, men of so much intelligence that they will hardly attribute to trade jealousy alone the objection which medical men have to the recommendation of 'sure cures' for baldness, fits, rupture, consumption, and so on, to persons who are apt to regard their religious teachers as safe guides in matters of health or disease; and who are not sufficiently familiar with the subtleties of the newspaper business to distinguish between the responsibilities of the editor and those of the publisher. As a fact most readers of periodicals have the impression that the advertisements they contain are endorsed by the editor. Advertisers rely upon this fact; and we cannot understand the casuistry which satisfies the conscience of a man who edits a periodical, ostensibly devoted to religion, which replenishes its coffers with the price of palpable falsehoods.

"If it were true that a religious newspaper could not be financially successful without taking money for the advertisement of worthless or delusive remedies, a course might be suggested worthy of the main object of these papers. But it is not true; for there are a few happy illustrations of the fact that, even in a religious newspaper, 'honesty is the best policy.'

"We call the attention of our large circle of readers to this matter, in the hope that they will use their influence to put an end to what we regard as a serious blemish in religious newspapers, and one which injures the good reputation which they ought to enjoy. And we call the attention of those religious newspapers to which our remarks may apply to this matter, in the hope that we shall not have to recur to it in a more explicit manner.

